# Regulatory ligand binding in plant chalcone isomerase–like (CHIL) proteins

**DOI:** 10.1016/j.jbc.2023.104804

**Published:** 2023-05-10

**Authors:** Emma R. Wolf-Saxon, Chad C. Moorman, Anthony Castro, Alfredo Ruiz-Rivera, Jeremy P. Mallari, Jason R. Burke

**Affiliations:** Department of Chemistry and Biochemistry, California State University San Bernardino, San Bernardino, California, USA

**Keywords:** chalcone isomerase–like, CHIL, DSF, flavonoid pathway, naringenin, tetrahydroxy chalcone, chalcone synthase

## Abstract

Chalcone isomerase–like (CHIL) protein is a noncatalytic protein that enhances flavonoid content in green plants by serving as a metabolite binder and a rectifier of chalcone synthase (CHS). Rectification of CHS catalysis occurs through direct protein–protein interactions between CHIL and CHS, which alter CHS kinetics and product profiles, favoring naringenin chalcone (NC) production. These discoveries raise questions about how CHIL proteins interact structurally with metabolites and how CHIL–ligand interactions affect interactions with CHS. Using differential scanning fluorimetry on a CHIL protein from *Vitis vinifera* (VvCHIL), we report that positive thermostability effects are induced by the binding of NC, and negative thermostability effects are induced by the binding of naringenin. NC further causes positive changes to CHIL–CHS binding, whereas naringenin causes negative changes to VvCHIL–CHS binding. These results suggest that CHILs may act as sensors for ligand-mediated pathway feedback by influencing CHS function. The protein X-ray crystal structure of VvCHIL compared with the protein X-ray crystal structure of a CHIL from *Physcomitrella patens* reveals key amino acid differences at a ligand-binding site of VvCHIL that can be substituted to nullify the destabilizing effect caused by naringenin. Together, these results support a role for CHIL proteins as metabolite sensors that modulate the committed step of the flavonoid pathway.

Flavonoids are a class of specialized metabolites that are ubiquitous across terrestrial land plants. The biological functions of flavonoids and their derivatives vary widely and include floral pigments, phytoalexins, structured scaffolds, and cellular signals (1, 2). In animals, dietary flavonoids are medically important antioxidants that also have beneficial procognitive and anti-inflammatory functions (3).

The flavonoid pathway in green plants begins with chalcone synthase (CHS), a type II polyketide synthase that catalyzes the condensation of one *p*-coumaroyl-CoA and three malonyl-CoAs into 2ʹ,4,4ʹ,6ʹ-tetrahydroxychalcone, also known as naringenin chalcone (NC). Downstream, chalcone isomerase (CHI) cyclizes NC to form 2*S*-naringenin (4, 5). The chalcone isomerase–like (CHIL) protein family is noncatalytic, yet it shares a “CHI fold” with CHI (6, 7). Genetic knockouts of CHIL reduce flavanol production in Japanese morning glory (*Ipomoea nil*) and *Arabidopsis thaliana* (8, 9). Overexpression of CHIL partially rescues the loss-of-function phenotype in *A. thaliana* and enhances flavanol production in *Dracaena cambodiana* (9, 10). CHIL-mediated flavanol production is upregulated *via* CHS binding, causing CHS to reduce production of a polyketide side product, *p*-coumaroyltriacetic acid lactone (CTAL) in favor of NC (11, 12, 13, 14). By rectifying the catalytic activity of CHS away from CTAL and toward NC, CHIL serves as a positive regulator of metabolic flux in the flavonoid pathway. Protein–enzyme binding between CHIL and CHS, as well as CHIL-induced CHS rectification, is conserved across diverse lineages of green plants (11). In *Humulus lupulus*, CHIL attenuates the production of two prenylated chalconoids, xanthohumol and demethylxanthohumol, into flavanols through direct binding and stabilization of the open-ring form of the metabolites (15). CHIL is the subject of two recent reviews with perspectives that frame these discoveries in the context of the flavonoid pathway, metabolon research, and the idea that CHIL may act as a sensor to regulate flux (16, 17). Despite recent findings, it is yet unknown how CHIL proteins interact structurally with metabolites. Additional research is needed to reveal the structural bases underlying the recently described functions of CHIL proteins.

Here, we present evidence of ligand selectivity in CHIL proteins and ligand-mediated regulation of CHIL–CHS complexes. Two CHILs are studied that represent distantly related species in the lineage of green plants: the lone CHIL from grape seed, *Vitis vinifera* (VvCHIL), and one of two known CHILs from the bryophyte, *Physcomitrella patens* (PpCHIL-A). Ligand-induced changes to protein *T*_m_s, or thermal shifts, reveal characteristics of CHIL binding to various metabolites possessing either chalcone, flavanone, or flavone-based scaffolds. Chalcones induce positive thermal shifts that are thermostabilizing for PpCHIL-A and VvCHIL. In addition, NC binding to CHIL increases the formation of CHIL–CHS complexes. Naringenin and quercetin decrease the thermostability of VvCHIL, and naringenin reduces protein–protein binding between VvCHIL and CHS. The effect of ligand-induced protein fold destabilization is similar to the studied example of Dwarf 14 (D14), a strigolactone receptor that undergoes a conformational change to facilitate signal transduction (18). These results suggest that CHILs may amplify or attenuate flux through the flavonoid pathway *via* metabolite-sensing mechanisms that regulate CHIL–CHS complexes.

To examine the structural bases for ligand-binding differences, high-resolution protein X-ray crystal structures of VvCHIL and PpCHIL-A are presented. A comparison of these structures reveals key amino acid differences at a protein surface pocket that canonically serves as an active site in related CHIs and a ligand binding site in fatty acid–binding proteins (FAPs) and fungal heme-binding proteins (7, 19, 20). Site-directed mutagenesis is used to exchange a key amino acid between VvCHIL and PpCHIL-A, resulting in a variant of VvCHIL that is no longer destabilized by naringenin binding, as well as a variant of PpCHIL-A possessing enhanced naringenin binding. Interestingly, mutations to the canonical active-site/ligand-binding pocket have very little effect on NC binding for VvCHIL, suggesting distinct binding sites for NC and naringenin. Together, these findings reveal how divergent structural attributes of CHIL proteins direct ligand selectivity and protein conformational stability to affect CHIL–CHS interactions. The ability of CHILs to act as receptors that sense and respond to downstream metabolite concentrations reveals a logic that is universal to committed steps of metabolic pathways.

## Results

### Chalcone binding to VvCHIL and PpCHIL-A

**Figure 1:**
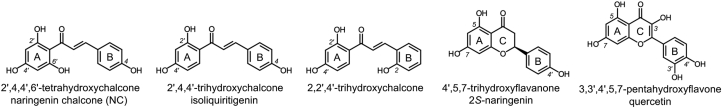
**Structures and names of molecules used in this study**.

Differential scanning fluorimetry (DSF) is a sensitive and label-free method for measuring the *T*_m_ of a protein, defined as the temperature at which 50% of a protein sample is unfolded (21). Positive *T*_m_ changes have been used to measure binding between a CHIL protein and chalcones (15). To elucidate a broader scope of CHIL–chalcone interactions, DSF was used to determine the thermal shifts that occur to VvCHIL and PpCHIL-A upon binding naturally occurring chalcones containing different substitution patterns (Fig. 1).

**Figure 2:**
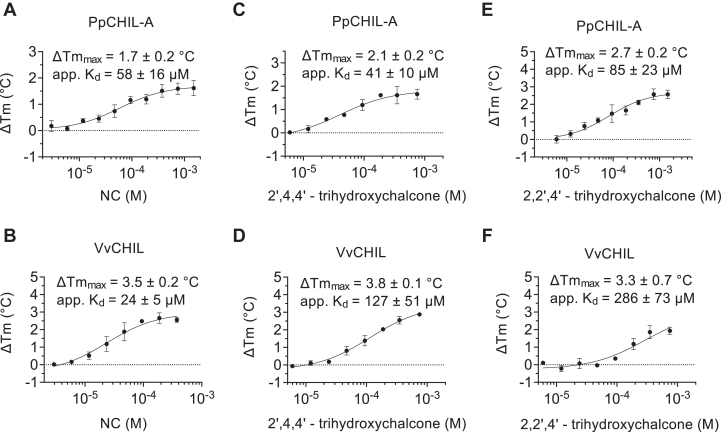
**CHIL–chalcone binding specificity.** The thermal shift response for binding of NC to (*A*) PpCHIL-A and (*B*) VvCHIL. The thermal shift response to 2ʹ,4,4ʹ-trihydroxychalcone for (*C*) PpCHIL-A and (*D*) VvCHIL. The thermal shift response to 2,2ʹ,4ʹ-trihydroxychalcone for (*E*) PpCHIL-A and (*F*) VvCHIL. Error bars are standard deviations of independent experiments (n = 4). CHIL, chalcone isomerase–like protein; NC, naringenin chalcone; PpCHIL, CHIL from bryophyte *Physcomitrella patens*; VvCHIL, CHIL protein from *Vitis vinifera.*

Titration of NC into PpCHIL-A produces a positive thermal shift, indicating a protein fold–stabilizing effect with a maximum thermal shift (ΔTm_max_) of 1.7 ± 0.2 °C. A single-site binding equation was used to fit the apparent equilibrium binding dissociation constant (apparent *K*_*d*_) of 58 ± 16 μM (Fig 2*A*). Because the apparent *K*_*d*_ values are derived by DSF, they do not account for the temperature dependence of the binding constant (22).

When the experiment is conducted using VvCHIL, NC binds with an apparent *K*_*d*_ of 24 ± 5 μM, producing a ΔTm_max_ of 3.5 ± 0.2 °C (Fig. 2*B*). The apparent *K*_*d*_ values for NC binding to VvCHIL and PpCHIL-A are similar to the reported *K*_*d*_ of 43 μM and ΔTm_max_ of 6.4 °C for HlCHIL1 from *H. lupulus*, measured by DSF (15). HlCHIL1 (A0A2U7XUH7) is a relatively distant ortholog of VvCHIL and PpCHIL-A and is more closely related to the family of FAPs, perhaps possessing functions different from other CHILs (15) (Fig. S1).

To determine which functional groups of NC are directly involved in binding to CHIL proteins, other naturally occurring chalcones were tested. The trihydroxy-substituted chalcones, 2ʹ,4,4ʹ-trihydroxychalcone (isoliquiritigenin) and 2,2ʹ,4ʹ-trihydroxychalcone, both possess a dihydroxy-substituted A ring, which differs from the trihydroxy-substituted A ring of NC (Fig. 1). In addition, 2,2ʹ,4ʹ-trihydroxychalcone has a hydroxyl at C2 on the B ring instead of at C4, as in NC, naringenin, and 2ʹ,4,4ʹ-trihydroxychalcone (Fig. 1). The results of the DSF experiments show that 2ʹ,4,4ʹ-trihydroxychalcone binds PpCHIL-A with an apparent *K*_*d*_ of 41 ± 10 μM and ΔTm_max_ of 2.1 ± 0.2 °C and VvCHIL with an apparent *K*_*d*_ of 127 ± 51 μM, with a ΔTm_max_ of 3.8 ± 0.1 °C (Fig. 2, *C* and *D*). The ligand, 2,2ʹ,4ʹ-trihydroxychalcone, binds PpCHIL-A with an apparent *K*_*d*_ of 85 ± 23 μM and ΔTm_max_ of 2.7 ± 0.2 °C and VvCHIL with an apparent *K*_*d*_ of 286 ± 23 μM and ΔTm_max_ of 3.3 ± 0.7 °C (Fig. 2, *E* and *F*).

The most significant outcome of this comparative set of experiments is that VvCHIL is selective for NC binding relative to other chalcones, whereas PpCHIL-A is not. This is supported by the finding that VvCHIL has approximately 5-fold and 11-fold smaller apparent *K*_*d*_ values for NC binding relative to 2ʹ,4,4ʹ-trihydroxychalcone and 2,2ʹ,4ʹ-trihydroxychalcone, respectively. These differences reveal components of a preferred binding epitope on chalcone ligands, which includes the 6ʹOH on the A ring and a hydroxyl at the C4 *versus* C2 position on the B ring. PpCHIL-A has lower selectivity for different chalcones such that it does not bind with an appreciable difference to NC or 2ʹ,4,4ʹ-trihydroxychalcone and has only a twofold greater apparent *K*_*d*_ for 2,2ʹ,4ʹ-trihydroxychalcone. Together, these results suggest that VvCHIL and PpCHIL-A may possess different structural elements for binding to chalcones and other related metabolites.

### Naringenin increases the thermostability of PpCHIL-A and decreases the thermostability of VvCHIL

**Figure 3:**
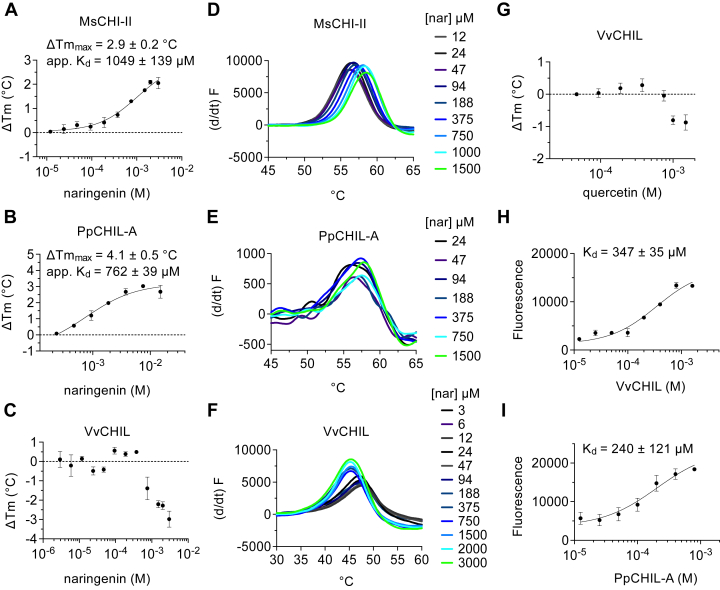
**Naringenin and quercetin binding effects on CHIL and CHI proteins.** DSF assays of CHI and CHIL–flavanol binding interactions for (*A*) MsCHI-II and naringenin (n = 4), (*B*) PpCHIL-A and naringenin (n = 2), and (*C*) VvCHIL and naringenin (n = 4). Examples of derivative fluorescence data from DSF assays that were used to calculate *T*_m_ values for (*D*) MsCHI-II and naringenin, (*E*) PpCHIL-A and naringenin, and (*F*) VvCHIL and naringenin. *G*, DSF assay of VvCHIL with quercetin (n = 4). *H*, fluorescence enhancement of quercetin in the presence of increasing concentrations of VvCHIL (n = 2), and (*I)*, increasing concentrations of PpCHIL-A (n = 2). Error bars are standard deviations of independent experiments. CHI, chalcone isomerase; CHIL, chalcone isomerase–like protein; DSF, differential scanning fluorimetry; MsCHI-II, type II CHI from *Medicago sativa*; PpCHIL-A, CHIL from bryophyte *Physcomitrella patens*; VvCHIL, CHIL protein from *Vitis vinifera*.

Naringenin is the product of NC cyclization by CHI and possesses a flavanone scaffold that is conformationally restricted relative to chalcones (Fig. 1). The protein X-ray crystal structure of naringenin bound to type II CHI from *Medicago sativa* (MsCHI-II) reveals the location and orientation through which naringenin binds to the enzyme active site (19). When MsCHI-II is heated in the presence of increasing concentrations of naringenin, the increase in *T*_m_ produces a ΔTm_max_ of 2.9 ± 0.2 °C and an apparent *K*_*d*_ of 1019 ± 115 μM (Fig. 3*A*). Ligand concentrations used are reported in Table S1. For PpCHIL-A, protein thermostability also increases with increasing naringenin, producing a ΔTm_max_ of 3.6 ± 0.3 °C and an apparent *K*_*d*_ of 762 ± 39 μM (Fig. 3*B*). These experiments reveal that PpCHIL-A is similar to MsCHI-II in the thermal shift produced by naringenin, suggesting that PpCHIL-A and MsCHI-II may have similar modes of binding to naringenin.

For VvCHIL, naringenin binding produces the opposite effect on the thermal shift relative to PpCHIL-A and MsCHI-II, resulting in reduced protein *T*_m_s at high naringenin concentrations (Fig. 3*C*). The loss of DSF signal at high naringenin makes the VvCHIL data unsuitable for quantitative analysis of ligand binding because the condition of saturation of ligand binding cannot be determined. The first derivative of the change in fluorescence of SYPRO orange dye was used to calculate the *T*_m_ under various conditions. From these data, it is apparent that the negative thermal shift of VvCHIL in the presence of high naringenin is different from the positive and dose-dependent thermal shifts of naringenin with MsCHI-II or PpCHIL-A (Fig. 3, *D*–*F*). Notably, the negative thermal shift of VvCHIL is comparatively switch like, capturing two distinct states (Fig. 3*F*). This suggests that a less-stable conformational ground state of the VvCHIL protein fold is favored upon naringenin binding.

Quercetin is relatively abundant in shoots and leaves of *V. vinifera* when compared with other flavonoids, such as naringenin (23, 24). In the flavonoid pathway, quercetin is made three steps downstream of naringenin. Quercetin differs from naringenin by additional hydroxyl groups on the B and C rings and an α,β-unsaturated carbonyl within the C ring (Fig. 1). Because of the relative abundance of quercetin in plants, we sought to determine whether quercetin also causes a fold-destabilizing effect when binding VvCHIL. Titrating quercetin into VvCHIL induces a negative thermal shift to the protein above 750 μM (Fig. 3*G*). Unfortunately, the DSF signal for the PpCHIL-A protein is very low relative to other proteins studied (Fig. 3*E*), and the intrinsic fluorescence of quercetin obstructs the weak fluorescence signal from the SYPRO orange dye in the DSF assay for this protein. Therefore, the effects of quercetin on PpCHIL-A by DSF were not measured. The intrinsic fluorescence of quercetin has been used to measure protein–ligand binding directly (25). Naringenin does not have similar fluorescent properties that would make it suitable for this assay (25). Binding-induced fluorescence enhancements of quercetin in the presence of increasing concentrations of CHIL were measured to determine *K*_*d*_ values of 347 ± 35 μM for VvCHIL and 240 ± 121 μM for PpCHIL-A (Fig. 3, *H* and *I*). The destabilization effect of quercetin on VvCHIL, as measured by DSF, is observed for quercetin concentrations between 750 μM and 1 mM (Fig. 3*G*). It is notable that the reduction in thermostability correlates closely with quercetin concentrations relevant for binding to VvCHIL, as measured by fluorescence (Fig. 3*H*); for example, based on the *K*_*d*_ value for VvCHIL, the fraction of VvCHIL bound to 750 μM quercetin is 0.68. Together, these measurements verify that binding occurs between quercetin and the CHIL proteins and reveal that quercetin destabilizes VvCHIL; however, the negative thermal shift to VvCHIL caused by quercetin is relatively small when compared with the negative thermal shift caused by naringenin (Fig. 3, *C* and *G*). From these findings, it is not straightforward to say whether quercetin binds to CHILs better or worse than chalcones. Because of differences in the binding assays needed for the different ligands, the equilibrium binding constants measured (*i.e.*, apparent *K*_*d*_ *versus K*_*d*_) do not have a known quantitative relationship.

### NC and naringenin affect CHIL–CHS complex formation

**Figure 4:**
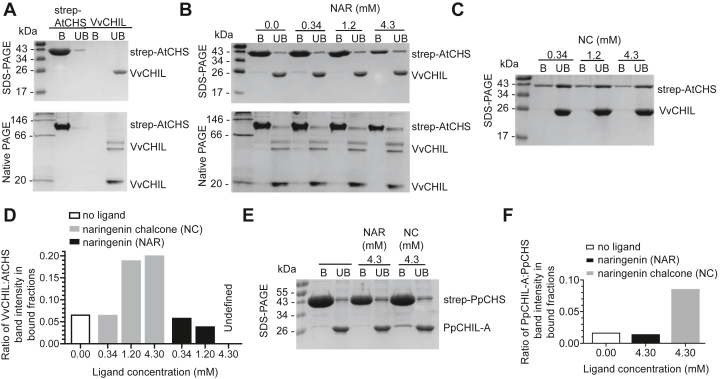
**Protein pull-down assays of CHIL and CHS.***A*, SDS-PAGE (*top*) and native PAGE (*bottom*) of StrepTactin-bound (*B*) and unbound (UB) fractions. Strep-AtCHS is observed primarily in the bound fraction because of the binding interaction between the strep-tag and the streptavidin-linked resin. Untagged VvCHIL does not bind to the resin and is observed only in the unbound fraction. *B*, pull-down assay results of mixing strep-AtCHS and VvCHIL together in the absence or in the presence of increasing concentrations of naringenin, (*C*) or increasing concentrations of naringenin chalcone (NC). *D*, quantification of the effects of ligands on VvCHIL–AtCHS binding. The ratio of intensities of VvCHIL to AtCHS bands from bound fractions of the SDS-PAGE gels was used to measure changes to complex formation caused by the presence of ligands. At 4.30 mM naringenin, a band for untagged VvCHIL was below the threshold of detection in the strep-AtCHS–bound fraction. The ratio of band intensities of untagged VvCHIL to strep-AtCHS is therefore undefined. *E*, pull-down assay results of mixing strep-PpCHS and PpCHIL-A together in the absence or in the presence of 4.3 mM naringenin and 4.3 mM NC. *F*, quantification of the effects of ligands on PpCHIL–PpCHS binding from the SDS-PAGE gel in *E*. AtCHS, CHS from *Arabidopsis thaliana*; CHIL, chalcone isomerase–like protein; CHS, chalcone synthase; PpCHS, CHS from *Physcomitrella patens*; VvCHIL, CHIL protein from *Vitis vinifera*.

Functional CHS–CHIL binding interactions are conserved throughout green plants and can be constituted using CHS and CHIL proteins from nondistant species (11). A protein pull-down method was devised using strep-tagged CHS; however, in our experiment, strep-tagged VvCHS failed to bind to the StrepTactin beads, so CHS from *A. thaliana* (strep-AtCHS) was used to capture untagged VvCHIL. Results of a binary protein-binding experiment show that strep-AtCHS binds to StrepTactin resin, whereas untagged VvCHIL does not (Fig. 4*A*). When strep-AtCHS and untagged VvCHIL are mixed together in the presence of StrepTactin resin, VvCHIL is observed in the bound fraction because of a direct binding interaction with strep-AtCHS (Fig. 4*B*). When the pull-down assay is conducted in the presence of increasing naringenin concentrations, VvCHIL–AtCHS binding is increasingly disrupted (Fig. 4*B*). Native PAGE shows that VvCHIL remains folded in the presence of all ligand concentrations tested, suggesting that the disruption to AtCHS–VvCHIL binding interaction may be due to a conformational change in the VvCHIL protein, rather than unfolding of the protein (Fig. 4*B*). In contrast to the effect seen with naringenin, the presence of NC enhances binding between strep-AtCHS and VvCHIL (Fig. 4*C*). The results of the protein pull-down experiments were quantified using densitometry (Figs. S2 and S3). It is notable that the band intensity of strep-AtCHS in the bound fraction decreases with increasing naringenin (Fig. 4*B*) and increasing NC (Fig. 4*C*). The effect of reduced strep-AtCHS binding to the StrepTactin resin in the presence of hydrophobic metabolites may be due to inhibited binding of the hydrophobic HPQ motif on the strep-tag to streptavidin-linked beads. Because the amount of untagged VvCHIL in the bound fraction is dependent upon the amount of strep-AtCHS in the bound fraction, bound VvCHIL was normalized. By taking the ratio of band intensities for VvCHIL to AtCHS for each of the StrepTactin-bound fractions on the SDS-PAGE gels, quantification shows that NC increases binding between VvCHIL and AtCHS, whereas naringenin has the opposite effect at 1.2 and 4.3 mM concentrations, decreasing the amount of VvCHIL that AtCHS can bind and pull down (Fig. 4*D*). Bound VvCHIL is present in bound fractions of non-native SDS-PAGE and native PAGE gels and in similar ratios to strep-AtCHS (Figs. 4*B* and S4). This analysis shows that VvCHIL protein in streptavidin-bound fractions is not significantly aggregated. When the experiment is conducted using a strep-tagged CHS from *P. patens* (strep-PpCHS) to capture untagged PpCHIL-A, PpCHIL-A is observed in the bound fractions for conditions without ligand present and with naringenin present (Fig. 4, *E* and *F*). When NC is present, bound PpCHIL-A is strongly enriched in the strep-PpCHS–bound fraction relative to the other conditions tested (Fig. 4, *E* and *F*). Together, these findings suggest a role for CHIL proteins as receptors with the capacity to upregulate the formation of CHIL–CHS complexes in the presence of NC, or alternatively in the case of VvCHIL, disrupt existing CHIL–CHS complexes in the presence of high naringenin. The significance of this is underscored by the work showing that CHIL proteins rectify CHS catalysis, thus affecting flux through the flavonoid pathway (11). These results suggest a model in which CHIL proteins can act as metabolic sensors that provide feedback to CHIL-assisted CHS catalysis at the rate-determining step of the flavonoid pathway.

### VvCHIL forms oligomers

**Figure 5:**
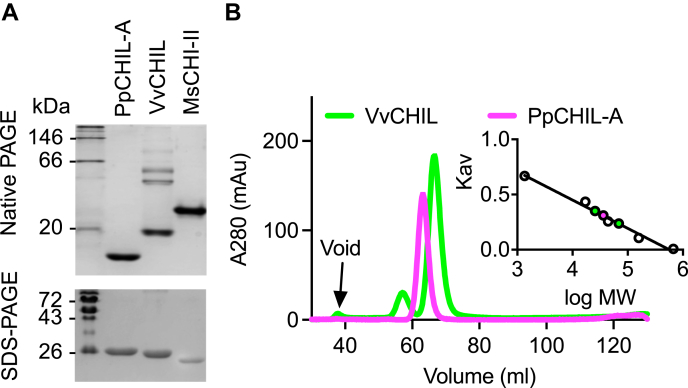
**Monomer and oligomer analysis of CHI and CHIL proteins.***A*, native PAGE (*top*) of PpCHIL-A, VvCHIL, and MsCHI-II. For comparison, the SDS-PAGE gel (*bottom*) shows the same proteins. *B*, size-exclusion chromatograms of VvCHIL and PpCHIL-A. VvCHIL elutes as two peaks, and PpCHIL-A elutes as a single peak. *Inlay*: a set of sizing standards used to generate a linear fit and how they compare with the calculated molecular weight values for VvCHIL (*green*) and PpCHIL-A (*magenta*). CHI, chalcone isomerase; CHIL, chalcone isomerase–like protein; MsCHI-II, type II CHI from *Medicago sativa*; PpCHIL, CHIL from bryophyte *Physcomitrella patens*; VvCHIL, CHIL protein from *Vitis vinifera.*

On native PAGE gels, VvCHIL displays two high–molecular weight bands indicative of oligomeric states and a single low–molecular weight band indicative of a monomer (Fig. 4*A*). Similar high–molecular weight bands are not observed on native PAGE gels for PpCHIL-A or MsCHI-II (Fig. 5*A*). Size-exclusion chromatography (SEC) of VvCHIL and PpCHIL-A reveals that VvCHIL elutes as two peaks and PpCHIL-A elutes as a single peak (Fig. 5*B*). When compared with a set of standard reference proteins, the single peak for PpCHIL-A elutes at a volume that calculates to a molecular weight of 36 kDa. The VvCHIL peaks calculate to 26 kDa (major peak) and 68 kDa (minor peak), indicating that a minor amount of VvCHIL is stable as a multimer, perhaps as a trimer in this experiment. For comparison, molecular weights of the monomeric forms of these proteins, calculated from their amino acid sequences, are 23 kDa for PpCHIL-A and 22 kDa for VvCHIL. Differences in SEC-based experimental molecular weight values relative to reference molecular weight values can be due to differences in the hydrodynamic radii of the protein molecules as they elute through a SEC column.

### Protein X-ray crystal structures of PpCHIL-A and VvCHIL

To enable a meaningful structural comparison of CHIL proteins, purified proteins were crystallized (Table 1). The protein X-ray crystal structures of PpCHIL-A and VvCHIL reveal CHI-type α/β folds with high structural homology to each other. The RMS value between the α-carbons within the PpCHIL-A and VvCHIL structures, a measure of structural identity, is 0.831 Å.

**Table 1:** X-ray crystallography data collection and refinement statistics

Crystal (PDB ID)	PpCHIL-A (8DLD)	VvCHIL (8DLC)
Data collection		
Space group	C121	P3_2_21
Cell dimensions		
*a*, *b*, *c* (Å)	62.55, 49.88, 76.82	49.4199, 49.4199, 170.851
*α*, *β*, γ (°)	90, 110.87, 90	90, 90, 120
Resolution (Å)	37.94–1.85	42.80–1.90
*R*_merge_	0.102 (0.667)	0.190 (1.6717)
*R*_pim_	0.049 (0.326)	0.050 (0.439)
CC ½	0.994 (0.812)	0.998 (0.815)
*I*/σ*I*	8.1 (2.1)	10.7 (2.2)
Completeness (%)	97.7	100.0
Multiplicity	5.3	15.2
Refinement		
Resolution (Å)	37.3–1.85 (1.916–1.85)	42.8–1.9 (1.968–1.9)
No. of unique reflections	18,703 (1837)	19,936 (1887)
*R*_work_/*R*_free_	0.1744 (0.3002)/0.2203 (0.3598)	0.1895 (0.4350)/0.2325 (0.4552)
No. of atoms	1794	1807
Protein	1635	1665
Water	159	142
*B*-factors (Å^2^)		
Average	37.03	30.14
Protein	36.54	29.45
Water	42.91	38.13
Rms deviations		
Bond lengths (Å)	0.011	0.011
Bond angles (°)	1.09	1.11
Ramachandran plot (%)		
Favored	99.51	98.05
Allowed	0.49	1.95
Outliers	0	0
PDB accession codes	8DLD	8DLC

Parentheses are for the highest resolution shell.

To date, there is no available structural evidence of CHIL proteins bound to ligands. Currently, the only other structure of a CHIL protein is from *A. thaliana* (AtCHIL) (7). The structure of AtCHIL revealed that CHILs possess a canonical CHI-fold surface pocket, which serves as an active site in CHIs and ligand-binding site in FAPs (7, 19). Motivated by the results of NC and naringenin binding to CHILs, we attempted to obtain ligand-bound structures of CHILs to determine the positions and orientations of ligand binding. Attempts to soak NC and naringenin into preformed crystals of VvCHIL and PpCHIL-A did not produce data with additional electron density near the potential ligand-binding site, and cocrystallization attempts were further unsuccessful. Analysis of apo structures of VvCHIL and PpCHIL-A reveal that both crystals contain symmetry-related protein molecules that form crystal contacts in and around the canonical CHI fold–binding pocket (Fig. S5).

**Figure 6:**
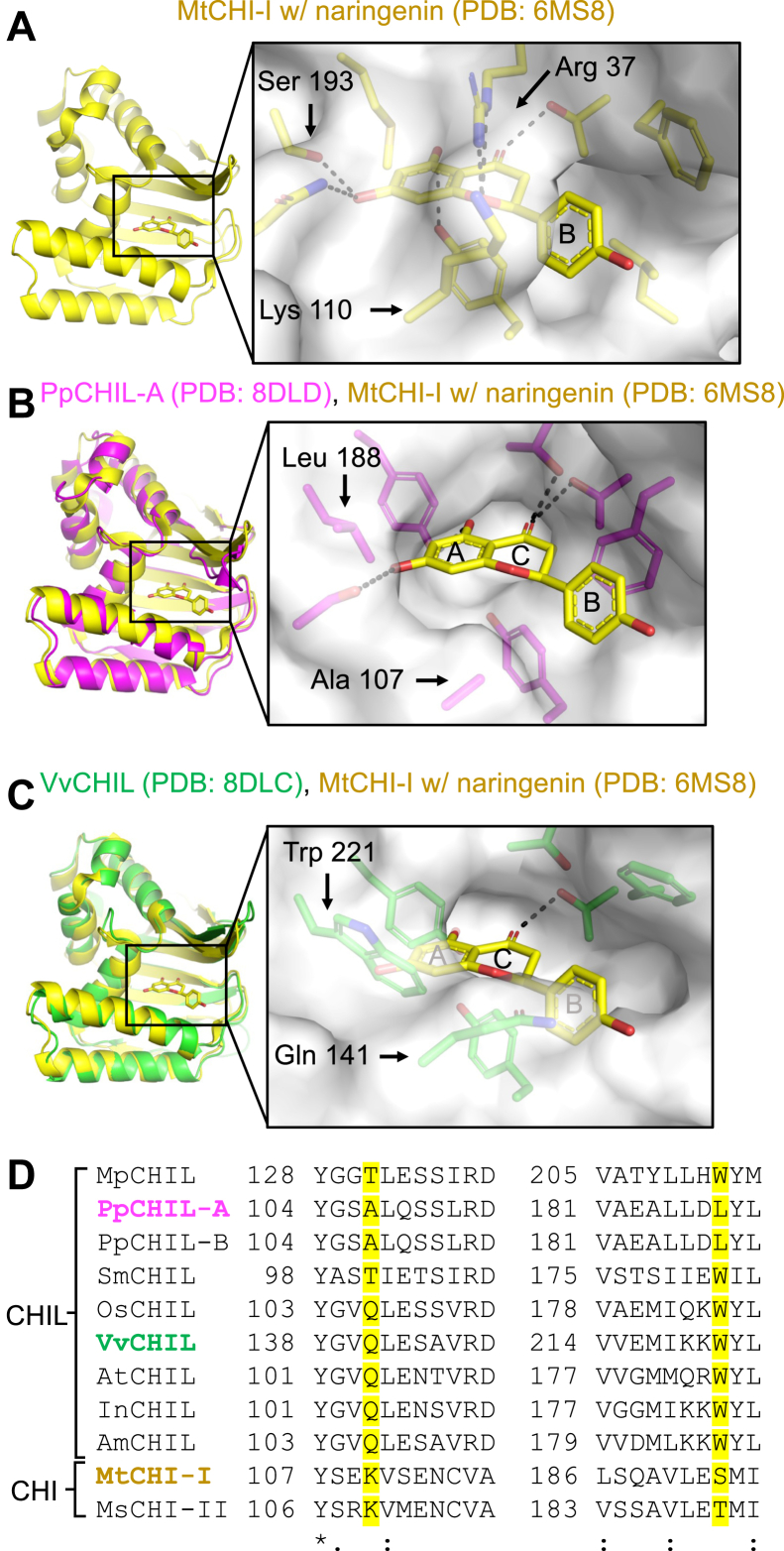
**Structural comparisons of PpCHIL-A and VvCHIL to MtCHI-I bound with naringenin.***A*, the product-bound structure of MtCHI-I shows naringenin bound and largely buried within the enzyme active site. *B*, the structure of PpCHIL-A structurally superimposed with MtCHI-I bound to naringenin. The superposition shows that naringenin (*yellow sticks*) fits within a pocket of PpCHIL-A (*magenta*), which is in a similar position to the active site of MtCHI-I. *C*, the structure of VvCHIL structurally superimposed with MtCHI-I bound to naringenin. The superposition suggests that naringenin (*yellow*) will not fit within the pocket of VvCHIL, which has a different shape than PpCHIL-A because of different amino acids. *D*, two segments from a multiple sequence alignment of CHIL and CHI amino acid sequences. The MSA contains CHILs from liverwort (*Marchantia polymorpha*, MpCHIL), bryophyte (*Physcomitrella patens*, PpCHIL-A and PpCHIL-B), lycophyte (*Selaginella moellendorffii*, SmCHIL), rice (*Oryza sativa*, OsCHIL), grapeseed (*Vitis vinifera*, VvCHIL), *Arabidopsis thaliana* (AtCHIL), Japanese morning glory (*Ipomoea purpurea*, IpCHIL), snapdragon (*Antirrhinum majus*, AmCHIL), and CHI enzymes from *Medicago truncatula* (MtCHI-I) and *Medicago sativa* (MsCHI-II). CHI, chalcone isomerase; CHIL, chalcone isomerase–like protein; MSA, multiple sequence alignment.

The apo PpCHIL-A and VvCHIL structures were compared for differences in potential ligand-binding sites to one another and to the published protein X-ray crystal structure of *Medicago truncatula* CHI-I (MtCHI-I) bound with naringenin (26). The structure of MtCHI-I bound to naringenin shows naringenin buried beneath an active site arginine (Arg 37) and lysine (K110) (Fig. 6*A*). This arginine is conserved across CHIs and related FAPs and is the principal catalytic component of CHIs; however, it is absent from all known CHIL proteins (7, 26, 27). PpCHIL-A and MtCHI-I have a high degree of structural similarity and align well to each other (C⍺-based RMS = 1.213 Å). This structural superposition reveals that PpCHIL-A contains a potential ligand-binding pocket that is analogous in some ways to the active site of MtCHI-I and complementary to the shape of naringenin, as it is oriented in the structure of MtCHI-I (Fig. 6*B*).

When the structures of VvCHIL and MtCHI-I bound with naringenin are aligned, a significant amount of the VvCHIL pocket is occluded (Fig. 6*C*). Most notably, tryptophan 221 (W221) and glutamine 141 (Q141) produce overlaps with naringenin. A multiple sequence alignment (MSA) of CHIL protein sequences was used to determine the degree of sequence conservation of these residues (Fig. 6*D*). Within the putative ligand-binding pocket, the tryptophan (W221 by VvCHIL numbering) is highly conserved across green plants with the notable exceptions of the two CHILs from *P. patens*, both of which possess a leucine in the same position (L188 by PpCHIL-A numbering). Nearby to the tryptophan in the structure of VvCHIL, a glutamine (Q141) also occupies the canonical binding site, yet this glutamine is not highly conserved across CHIL sequences (Fig. 6, *C* and *D*). In summary, the structures of VvCHIL and PpCHIL-A suggest that PpCHIL-A may possess a binding pocket that is suitable to accommodate naringenin. This structural hypothesis is consistent with the observation that naringenin binding to PpCHIL-A has a stabilizing effect, which is similar to MsCHI-II (Fig. 3, *A* and *B*). On the other hand, the presence of the tryptophan and glutamine side chains create a fundamentally different chemical topography for the VvCHIL-binding pocket, potentially changing the nature of ligand interactions. Supporting this idea, analysis of the VvCHIL-binding pocket reveals a pocket volume of 20.8 A^3^, whereas the volume of the PpCHIL-A–binding pocket is significantly larger, calculated to be 50.7 A^3^ (Fig. S6).

Briefly, it should be mentioned that at the time of submission of this article, structure predictions by AlphaFold (DeepMind) were made available for all protein sequences in the “protein universe” (28). The X-ray crystal structures of PpCHIL-A and VvCHIL were not available through the Protein Data Bank (PDB) prior to release of the AlphaFold predictions, and indeed, the PpCHIL-A X-ray crystal structure shows significant positional deviations when compared with the AlphaFold-predicted structure. A significant error in the AlphaFold model occurs in the β-hairpin motif abutting the ligand-binding site, which has significance in understanding protein–ligand interactions (Fig. S7). It has been noted that perils of AlphaFold include that naïve structure interpretations can lead to erroneous hypotheses (29). This result illustrates one example in which a confident AlphaFold structure prediction fails to accurately predict a protein ground state and should be used with caution.

### Amino acid substitutions to VvCHIL and PpCHIL-A

**Figure 7:**
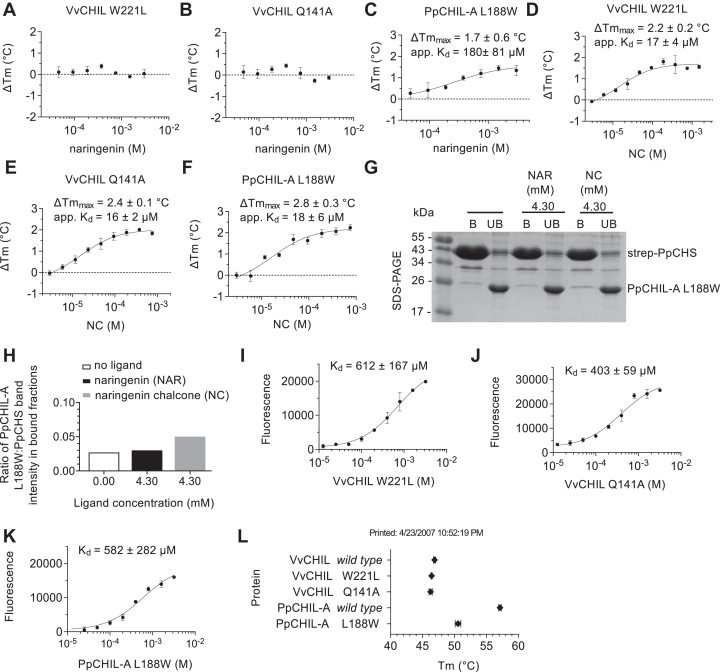
**Effects of amino acid substitutions on the ligand-binding properties of VvCHIL and PpCHIL-A.** DSF assays of CHIL–ligand binding interactions for (*A*) VvCHIL W221L and naringenin (n = 4), (*B*) VvCHIL Q141A and naringenin (n = 4), (*C*) PpCHIL-A L188W and naringenin (n = 2), (*D*) VvCHIL W221L and NC (n = 4), (*E*) VvCHIL Q141A and NC (n = 4), and (*F*) PpCHIL-A L188W and NC (n = 4). Error bars are standard deviations of independent experiments. *G*, SDS-PAGE of StrepTactin-bound (B) and unbound (UB) fractions from a protein pull-down experiment between strep-PpCHS and PpCHIL-A (untagged) in the presence of no ligand, 4.3 mM naringenin, or 4.3 mM NC. *H*, quantification of the effects of ligands on PpCHS–PpCHIL-A binding. The ratio of intensities of protein bands from bound fractions of the SDS-PAGE gels was used to measure changes to complex formation caused by the presence of ligands. Fluorescence enhancement assay of quercetin binding to (*I*) VvCHIL W221L (n = 2), (*J*) VvCHIL Q141A (n = 2), and (*K*) PpCHIL-A L188W (n = 2). *L*, *T*_m_ values of unliganded wildtype and variant CHIL proteins (n = 4). Error bars are standard deviations of independent experiments. CHIL, chalcone isomerase–like protein; DSF, differential scanning fluorimetry; NC, naringenin chalcone; PpCHIL, CHIL protein from the bryophyte, *Physcomitrella patens*; VvCHIL, CHIL protein from *Vitis vinifera*.

The comparison of VvCHIL and PpCHIL-A protein crystal structures suggests that W221 may be responsible for the destabilizing effect to VvCHIL caused by naringenin binding. Amino acid substitutions were made to the potential ligand-binding pockets, which exchange the tryptophan of VvCHIL (W221) for the positional-equivalent leucine in PpCHIL-A (L188), and vice versa. With the W221L substitution in place, VvCHIL does not undergo thermal destabilization in the presence of naringenin (Fig. 7*A*). Therefore, W221 may be required to facilitate the ligand-induced change to the less stable form of VvCHIL, which is observed in the presence of high naringenin (Fig. 3*C*). Similarly, a variant of VvCHIL with the Q141A substitution does not undergo thermal destabilization in the presence of naringenin, suggesting W221 and Q141 together are required to facilitate destabilization (Fig. 7*B*). The reciprocal substitution in PpCHIL-A, L188W, increases the apparent binding affinity for naringenin by approximately fourfold over wildtype PpCHIL-A while also reducing the effect of thermal stabilization by naringenin by approximately 2 °C (Fig. 7*C*).

To determine whether NC and naringenin bind to similar locations on VvCHIL and PpCHIL-A, we measured NC binding to the CHIL variants. Both W221L and Q141A bind to NC with an apparent *K*_*d*_ similar to wildtype VvCHIL (Figs. 2*B* and 7, *D* and *E*). These results reveal that the W221L and Q141A substitutions do not alter the NC binding interface on VvCHIL. PpCHIL-A differs from VvCHIL in this respect, since L188W increases the binding affinity of NC approximately threefold over wildtype PpCHIL-A (Figs. 7*F* and 2*A*).

When protein pull-down experiments were used to evaluate changes in complex formation of PpCHS–PpCHIL-A containing the PpCHIL-A L188W mutation, it was found that PpCHS pulls down PpCHIL-A L188W similarly in the presence and absence of naringenin; however, the binding effect is enhanced in the presence of 4.3 mM NC (Fig. 7, *G* and *H*). This is similar to the results observed for wildtype PpCHIL-A (Fig. 4, *E* and *F*), although the binding of PpCHIL-A L188W to PpCHS is reduced relative to wildtype in the presence of 4.3 mM NC (Figs. 7*H* and 4*F*). Together, these results suggest that changes to ligand binding caused by L188W in PpCHIL-A may not have dramatic effects on complex formation.

We examined the effects of the same amino acid substitutions on quercetin binding. Quercetin binds approximately twofold less to W221L than wildtype VvCHIL (Figs. 7*I* and 3*H*). Similarly, L188W reduces the binding affinity of quercetin for PpCHIL-A approximately twofold relative to wildtype (Figs. 7*J* and 3*I*). For the Q141A substitution, binding is not significantly altered relative to wildtype VvCHIL (Figs. 7*K* and 3*H*). These results show that quercetin binds at or near to the canonical CHI fold–binding pocket; specifically, near to W221 in VvCHIL or L188 in PpCHIL-A, and similar to naringenin binding to these proteins. The observation that L188W increases the apparent binding affinity for naringenin and decreases the binding affinity for quercetin may be due to differences in the structures of the metabolites and binding modes of the molecules to the protein pocket.

In consideration of the possibility that amino acid substitutions to VvCHIL and PpCHIL-A induce conformational changes, causing long-range effects that alter ligand binding, we examined the effects that the amino acid substitutions have on the *T*_m_s of the proteins. Relative to wildtype VvCHIL (*T*_m_ = 46.9 ± 0.2 °C), there is no significant change caused by W221L (*T*_m_ = 46.4 ± 0.2 °C) or Q141A (*T*_m_ = 46.3 ± 0.3 °C) (Fig. 7*L*). However, relative to wildtype PpCHIL-A (*T*_m_ = 57.2 ± 0.2 °C), a significant reduction in the protein *T*_m_ is caused by L188W (*T*_m_ = 50.6 ± 0.5 °C) (Fig. 7*L*). The *T*_m_ reduction in L188W induces a shift to a less-stable conformation or conformational ensemble of PpCHIL-A. This is consistent with the observation that L188W is sufficient to alter the binding of NC, naringenin, and quercetin and reduce the positive effect of NC on PpCHS–PpCHIL-A complex formation (Figs. 4, *E* and *F*, and 7, *G* and *H*). In terms of VvCHIL, the ligand-binding results show that chalcones bind to the protein surface in a location that is different from flavones and the canonical CHI fold–binding pocket.

## Discussion

The results presented here build upon recent findings that CHIL proteins modulate CHS activity (11, 12, 13, 14, 15), by providing evidence that CHILs are affected by metabolites in the flavonoid pathway. For VvCHIL, naringenin causes a fold-destabilizing effect (a decrease in *T*_m_) that disrupts CHIL–CHS binding. In general, fold-destabilizing effects caused by protein-ligand interactions are less commonly characterized than stabilizing effects and have been attributed to nonspecific causes such as interactions between ligands and unfolded protein states and changes to the ionic strength of the solution caused by hydrophobic ligands (30). It is clear that the effects observed here are due to specific protein–ligand interactions, since stabilizing *versus* destabilizing effects are specific to different CHIL proteins and are changeable through amino acid substitutions. Furthermore, precedent exists for ligand-induced fold destabilization as a signaling mechanism. The plant hormone strigolactone-related molecule, G24, binds and destabilizes the protein receptor, D14, and the less thermostable D14 conformation recruits a polyubiquitinated form of D53 and an F-box protein to promote D53 degradation (18, 31, 32, 33, 34). In a similar way, VvCHIL may be subject to regulation by the downstream metabolite, naringenin. However, the low millimolar concentration of naringenin required for the fold-destabilization effect to VvCHIL *in vitro* may not represent conditions *in vivo*. It is notable that the concentrations used for metabolite binding assays and pull-down assays may be greater than what have been measured as physiological in some cases (23, 24). However, quercetin and derivatives have been measured up to 420.4 mg/kg dry weight in a grapevine shoot cultivar (23). The flavonoid metabolon comprises CHS, CHIL, and CHI, among other enzymes, and metabolons concentrate and compartmentalize enzymes and metabolites within specific regions (11, 12, 13, 14, 15, 35, 36, 37, 38, 39, 40, 41). The VvCHIL protein shows a tendency for multimerization, which may play a role in metabolon regulation since it is known that CHS enzymes preferentially form homodimers (42, 43, 44). However, a CHIL–CHS monomer–monomer interaction has been reported in a cell-free system using chemical crosslinking (11). Taken together, the effect of naringenin in destabilizing VvCHIL and disrupting CHIL–CHS binding may constitute a signaling mechanism that functions to attenuate flavonoid pathway flux under conditions in which downstream products accumulate to critical concentrations.

Regulatory ligand binding in CHIL proteins raises the question of whether the evolutionary-related enzyme, CHI, is also subject to kinetic regulation through metabolite binding. Flavonoid pathway metabolites that are generated downstream of CHI, 5-hydroxy and 5-deoxy flavonoids and isoflavonoids, have been shown to act as competitive inhibitors of CHI enzymes (45), and through product inhibition, naringenin is also a known inhibitor of CHI (4). Thus, several potential negative feedback mechanisms into upstream enzymes in the flavonoid pathway are known.

Opposite to the effects of naringenin on VvCHIL, NC binds and stabilizes the VvCHIL and PpCHIL-A protein folds (increases the *T*_m_), and this promotes greater binding with CHS in protein–protein binding assays. These findings suggest two functional possibilities for CHIL–NC interactions. First, CHILs may act as NC receptors that amplify flavonoid pathway output by upregulating the formation of CHIL–CHS complexes in the presence of NC. This positive feedback loop would further favor the production of NC over the CHS-catalyzed side product, CTAL. On the other hand, it is not clear from this study if CHIL–CHS interactions promoted by NC are more productive in the types of catalytic rectification and enhancements that have been reported for these complexes (11). Therefore, it is a possibility that NC binding to CHIL attenuates flux through the flavonoid pathway in plants. Notably, NC binding to a CHIL protein from *H. lupulus*, HlCHIL1, has previously been demonstrated; however, HlCHIL1 is more closely related in sequence to the family of FAP proteins than CHIL proteins, and the significance of this protein–ligand interaction has not been studied (15).

*P. patens* has two genes that encode CHIL proteins but does not possess a gene that encodes a CHI (46). It has been postulated that CHILs may direct spontaneous ring closure reactions in a stereoselective manner to produce 2*S*-naringenin, rather than 2*R*-naringenin, in a way facilitating the stereochemical outcome catalyzed by CHI (17). Therefore, in the absence of a CHI enzyme, it makes sense that CHILs of *P. patens*, in particular, might serve as scaffolds for stereoselective NC cyclization, perhaps within the context of CHIL–CHS complexes. Notably, the stabilizing effects of naringenin binding to PpCHIL-A differ from the destabilization effects to VvCHIL; however, naringenin–PpCHIL-A binding is similar in several ways to what we observe for the CHI enzyme, MsCHI-II. The protein X-ray crystal structure of PpCHIL-A reveals a binding pocket that is analogous in some ways to the active site of MtCHI-I, and molecular dynamics simulations have revealed that the active-site architecture of MtCHI-I directs stereoselective ring closure of NC to form 2*S*-naringenin instead of 2*R*-naringenin (26). Therefore, together, these observations suggest that the naringenin-binding characteristics of *P. patens* CHIL-A may underlay a second function for these CHILs, which perhaps is unique among CHIL proteins of green plants.

The results presented here do not pinpoint exactly where or how NC and naringenin bind to VvCHIL or PpCHIL-A, yet it is clear for VvCHIL that the two metabolites bind to distinct interfaces, possibly overlapping. Variations in CHI–CHIL ligand binding have been revealed by protein X-ray crystal structures, and these examples may be informative in the context of the findings presented here. The DaCHI1 enzyme from *Deschampsia antarctica* binds to 2ʹ,4,4ʹ-trihydroxychalcone (isoliquiritigenin) in an orientation that is relatively solvent exposed and mostly set aside from the conical CHI fold–binding pocket (PDB ID: 5YX4) (47). A similar mode of binding of NC to VvCHIL might explain why the W221L and Q141A substitutions do not alter the apparent binding affinity of NC for VvCHIL. Likewise, a phylogenetic reconstruction of a CHI–CHIL ancestor protein (PDB ID: 5WKR) and a variant from a directed evolution experiment containing only nascent CHI catalytic activity (PDB ID: 5WKS), both reveal naringenin binding in an alternate orientation that is somewhat removed from the canonical CHI-fold pocket (27). In the context of the data presented, these structures provide insights into possible ways in which NC and naringenin may bind to regulate CHIL proteins.

## Experimental procedures

### Gene analysis, cloning, site-directed mutagenesis, and MSA

Synthetic CHIL and CHS genes were codon optimized for *Escherichia coli* and produced by Genscript. Gene-specific primers were designed to subclone each gene into a modified pET vector with an N-terminal 8 histidine affinity tag and thrombin cleavage site (Table S2). Site-directed mutagenesis of VvCHIL was performed using a modified QuikChange protocol (48). An MSA was performed using the ClustalO online server with maximum default iterations (49). The alignment contains the following proteins: *Marchantia polymorpha*, MpCHIL (*Marchantia polymorpha* CHIL; A0A176VJG8); *P. patens*, PpCHIL-A (A9T3E4); *P. patens*, PpCHIL-B (A9SRW1); *Selaginella moellendorffii*, SmCHIL (*Selaginella moellendorffii* CHIL; D8QX37); *Oryza sativa*, OsCHIL (*Oryza sativa* CHIL; Q2RBC7); *V. vinifera*, VvCHIL (A0A438J2L0); *A. thaliana*, AtCHIL (Q8VZW3); *Ipomoea purpurea*, IpCHIL (*Ipomoea purpurea* CHIL; X5IGL5); *Antirrhinum majus*, AmCHIL (*Antirrhinum majus* CHIL; W0SN99), *M. truncatula*, MtCHI-I (B7FJK3); and *M. sativa*, MsCHI-II (P28012).

### Protein expression and purification

The expression plasmids containing *P. patens* A CHIL (PpCHIL-A) (A9T3E4), *V. vinifera* CHIL (VvCHIL) (A0A438J2L0), and *M. sativa* CHI-II (MsCHI-II) (P28012) were transformed into BL21 DE3-pRIL cells. Cells were grown in Terrific broth and induced overnight at 20 ºC. His-tagged proteins were purified by nickel-affinity chromatography, using a buffer containing 150 mM NaCl, 1 mM 2-mercaptoethanol, and 25 mM Tris–HCl (pH 8.0). Following purification, the polyhistidine 8 tag was removed overnight at 4 °C with thrombin. The cut CHIL proteins were dialyzed overnight at 4 °C and then purified again over a nickel-affinity column by collecting the flow-through. Strep-tagged *A. thaliana* CHS (P13114) and *P. patens* CHS (Q2VAZ3) were transformed into BL21 DE3-pRIL cells, grown in Terrific broth, and induced overnight at 20 ºC. The protein was purified on StrepTactin Sepharose resin and then further purified by anion exchange on a Fast Flow agarose Q column.

### SEC

SEC was performed on a HiPrep Sephacryl S-200 high-resolution column run on an AKTA explorer FPLC. The column was equilibrated and eluted at a rate of 1 ml/min with a buffer containing 200 mM NaCl, 25 mM Tris (pH 8.0), and 2 mM DTT. Purified CHIL proteins with the His tags removed were used for analysis. Molecular weight reference standards were generated using vitamin B12, myoglobin (horse), ovalbumin (chicken), gamma globulin (bovine), and thyroglobulin (bovine), available as a broad molecular weight marker reference set (Bio-Rad).

### Synthesis of NC

2ʹ,4,4ʹ,6ʹ-tetrahydroxychalcone (NC) was prepared by treating 1.00 g of naringenin (Sigma–Aldrich) with 5% sodium hydroxide in 50 ml methanol. The mixture was refluxed in a 100 ml round bottom flask at 70 °C for 1 h. The reaction mixture was added to 200 ml of 2 M hydrochloric acid in a 1 l separatory funnel. The aqueous layer was extracted with ethyl acetate (1 × 150 ml, 2 × 100 ml), and then the combined ethyl acetate layers were washed with water (3 × 100 ml) and washed with brine (2 × 100 ml). The ethyl acetate was dried over anhydrous sodium sulfate, and then the solvent was removed using rotary evaporation. This resulted in 0.6 g of crude product. NC preparation was initially confirmed using TLC run with 90% chloroform: 10% methanol. Flash chromatography was performed on 200 to 400 mesh silica flash columns (Grace/Buchi) using a Reveleris X2 chromatography instrument (Grace/Buchi). Naringenin and NC were resolved using a linear gradient of 80% hexane/20% ethyl acetate to 100% ethyl acetate. Chromatography solvents were obtained through Fisher Scientific. ^1^H and ^13^C data were collected on a JEOL NMR instrument (400 MHz). ^1^H NMR (CD_3_OD) (400 MHz): δ8.04 (1H, d, *J* = 15.6 Hz), 7.66 (1H, d, *J* = 15.6 Hz), 7.46 (2H, d, *J* = 7.6 Hz), and 6.78 (2H, d, *J* = 7.6 Hz), 5.82 (2H, s). ^13^C NMR (CD_3_OD): δ194.13, 166.16, 165.97, 160.97, 143.64, 131.29, 128.47, 125.53, 116.80, 105.88, and 95.98.

### Quercetin fluorescence assay

Fluorescence enhancement of quercetin (Thermo Scientific) was measured in the presence of VvCHIL and PpCHIL-A. The assays were performed in a buffer containing 25 mM Tris (pH 8.0), 150 mM NaCl, and 2% dimethyl sulfoxide (DMSO), used to solubilize quercetin. Protein concentrations were varied from 4.3 to 2250 μM relative to a constant concentration of quercetin (10 μM). After a binding equilibration period of 10 min at 25 °C, changes to the relative fluorescence intensity of quercetin (excitation 485 nm and emission 545 nm) were measured using a Vector X3 multimode plate reader (PerkinElmer). Background fluorescence from identical samples not containing quercetin was subtracted, and change-in-fluorescence data were fit using GraphPad Prism 9 (GraphPad Sofware, Inc) to a single-site binding equation in order to determine equilibrium dissociation constant (*K*_*d*_) values.

### DSF

Assays were performed using SYPRO orange dye (1:625 dilution) and 6 μM CHIL protein against dilution series of (2*R*/2*S*)-naringenin (Sigma–Aldrich), NC, 2ʹ,4,4ʹ-trihydroxychalcone (Indofine), 2,2ʹ,4ʹ-trihydroxychalcone (Indofine), and quercetin (Thermo Scientific). Reactions were run in 20 μl reaction volumes and in a buffer containing 150 mM NaCl, 25 mM Tris (pH 8.0), 2 mM DTT, and 2% DMSO. Because of low solubility, 2% DMSO was used for all reactions to enhance ligand solubility. Experiments were performed on a QuantStudio 3 Real-Time PCR and analyzed using Protein ThermalShift software (Thermo Fisher Scientific). Replicates of two to four experiments were conducted for each condition, and GraphPad Prism 9 was used to fit maximum thermal shift (ΔTm_max_) and apparent *K*_*d*_ values.

### Protein pull-down assay

Protein pull-down assays were conducted using 1 mg of purified strep-tagged AtCHS or PpCHS and 0.3 ml StrepTactin Sepharose resin. The untagged purified VvCHIL or PpCHIL-A proteins was used as bait (0.5 mg). Binding reactions were incubated at room temperature either without ligand and 2% DMSO, or with the reported concentration of ligand and 2% DMSO. The StrepTactin resin was washed 4× with 1 ml of buffer in order to remove unbound protein prior to elution. Each wash buffer contained the reported concentration of ligand, 2% DMSO, 150 mM NaCl, 50 mM Tris (pH 8.0), and 2 mM DTT. Strep-CHS was eluted from the StrepTactin resin with 0.1 ml elution buffer, which contains 1 mM desthiobiotin, 2% DMSO, 150 mM NaCl, 50 mM Tris (pH 8.0), and 2 mM DTT. The pull-down assays were resolved using 15% acrylamide SDS-PAGE and native PAGE gels and imaged and quantified using Image Lab (Bio-Rad). About 50 mM of DTT was used in the native PAGE loading buffer.

### Protein crystallization, data collection, and processing

PpCHIL-A and VvCHIL proteins were expressed as described previously and further purified *via* ion-exchange chromatography using a resource Q, followed by SEC on a Superdex 200 column with a buffer containing 200 mM NaCl, 25 mM Tris–HCl (pH 8.0), and 2 mM DTT. Proteins were concentrated to 20 mg/ml and screened broadly over 600 conditions. Purified PpCHIL-A crystallized after approximately 1 week in a condition containing 30% Peg 4K and 0.2 M ammonium sulfate. Purified VvCHIL crystallized after 1 week in a condition containing 20% (w/v) PEG 3350 and 200 mM potassium nitrate. Crystals were transferred to a cryogenic stabilizing solution containing an additional 20% glycerol and flash frozen in liquid nitrogen. X-ray diffraction data were indexed and integrated with iMOSFLM (50) and scaled with POINTLESS (51) in space groups C121 for PpCHIL-A and P3_2_21 for VvCHIL. For PpCHIL-A and VvCHIL, molecular replacement solutions were obtained using PHASER (52) and the search model (PDB ID: 4DOK) (7). The atomic models were built through iterative rounds of manual modifications in COOT (53), and refinement with PHENIX (54), according to default parameters. Protein crystal structure solutions are deposited in the PDB as 8DLC for VvCHIL and 8DLD for PpCHIL-A.

## Data availability

Data corresponding to protein X-ray crystallography experiment data have been deposited in a publicly accessible database, PDB (VvCHIL, 10.2210/pdb8dlc/pdb; PpCHIL-A, 10.2210/pdb8dld/pdb). All other data are contained within the article and supporting information.

## Supporting information

This article contains supporting information (28, 49, 55, 56).

## Conflict of interest

The authors declare that they have no conflicts of interest with the contents of this article.
