# Supplementary material for: Regulatory ligand binding in plant chalcone isomerase–like (CHIL) proteins
Source: J Biol Chem. 2023 May 10;299(6):104804. doi: 10.1016/j.jbc.2023.104804 (PMC10276294; doi:10.1016/j.jbc.2023.104804)
Supplement: Supporting information [file mmc1.docx]

**Supporting Information**

**Regulatory ligand binding in plant chalcone isomerase-like (CHIL) proteins.**

Emma R. Wolf-Saxon^1^, Chad C. Moorman^1^, Anthony Castro^1^, Alfredo Ruiz-Rivera^1^, Jeremy P. Mallari^1^ and Jason R. Burke^1*^

^1^Department of Chemistry and Biochemistry, California State University San Bernardino, San Bernardino, California 92407, USA.

*Corresponding Author:

Jason R. Burke

Email: [jason.burke@csusb.edu](mailto:jason.burke@csusb.edu)

Phone: +1-909-537-5338

| **Figure** | **3A** | **3B** | **3C** | **3G** | **3H** | **3I** |
| --- | --- | --- | --- | --- | --- | --- |
| Protein | MsCHI-II | PpCHIL-A | VvCHIL | VvCHIL | VvCHIL | PpCHIL-A |
| Ligand | naringenin | naringenin | naringenin | quercetin | quercetin | quercetin |
| Y-axis value | ΔTm (°C) | ΔTm (°C) | ΔTm (°C) | ΔTm (°C) | fluorescence | fluorescence |
| X-axis value | naringenin (M) | naringenin (M) | naringenin (M) | quercetin (M) | VvCHIL (M) | PpCHIL-A (M) |
| Concentration used (M) | 1.20E-05 | 2.34E-04 | 2.94E-06 | 4.70E-05 | 0.00E+00 | 0.00E+00 |
|  | 2.40E-05 | 4.69E-04 | 5.88E-06 | 9.40E-05 | 1.25E-05 | 1.25E-05 |
|  | 4.70E-05 | 9.38E-04 | 1.18E-05 | 1.88E-04 | 2.50E-05 | 2.50E-05 |
|  | 9.40E-05 | 1.87E-03 | 2.40E-05 | 3.75E-04 | 5.00E-05 | 5.00E-05 |
|  | 1.88E-04 | 3.75E-03 | 4.70E-05 | 7.50E-04 | 1.00E-04 | 1.00E-04 |
|  | 3.75E-04 | 7.50E-03 | 9.40E-05 | 1.00E-03 | 2.00E-04 | 2.00E-04 |
|  | 7.50E-04 | 1.50E-02 | 1.88E-04 | 1.50E-03 | 4.00E-04 | 4.00E-04 |
|  | 1.50E-03 |  | 3.75E-04 |  | 8.00E-04 | 8.00E-04 |
|  | 2.00E-03 |  | 7.50E-04 |  | 1.60E-03 |  |
|  | 3.00E-03 |  | 1.50E-03 |  |  |  |
|  |  |  | 2.00E-03 |  |  |  |
|  |  |  | 3.00E-03 |  |  |  |

**Table S1. Ligand concentrations used for binding experiments shown in Fig 3.**

| **Name** | **Forward Primer** |
| --- | --- |
| PpCHIL-A | gctaggcatgctgaattcggtccgcaggttg |
| VvCHIL | gctaggcatgctgaattcggcaccgaaatgg |
| Pp_L188W_F | ctgctggactggtacctgggtgaaaatacc |
| Vv_W221L_F | gatcaaaaaattgtacctgtccggtacgaaagg |
| Vv_Q141A_F | gtatggtgtggcactggaatccgctgttcg |
| Vv_CHS_F | ggcatgctgaattctggtcccatccacaatttgaaaaggggagcatggcttctgctgggg |
| Pp_CHS_F | ggcatgctgaattctggtcccatccacaatttgaaaaggggagcatggtgtcagtggggg |
|  | **Reverse Primer** |
| PpCHIL-A | gctaggcatgctgcggccgctcatgccgcaatcgc |
| VvCHIL | gctaggcatgctctcgagtcatttggacagttcg |
| Pp_L188W_R | cccaggtaccagtccagcagggcttccgcg |
| Vv_W221L_R | cggacaggtacaattttttgatcatttcgacaac |
| Vv_Q141A_R | ggattccagtgccacaccatactgactgcc |
| Vv_CHS_R | gctaggcatgctctcgagttacttaagtgtgaaaggagag |
| Pp_CHS_R | gctaggcatgctctcgagtcaagcggagttgggagcggcgcgg |

**Table S2. DNA primers used in this study.**

pid  **1** **[ . . . . :** **50**

1 VvCHIL 100.0% **MGLNISCTLIFLCDSSLNISFLLLLHHLCSPPPNPQPVMGTEMVMVDEIP**

2 PpCHILA 42.7% **--------------------------------------MGPQVVKVEDID**

3 HlCHIL1 14.5% **-----------------------MATALNS--KNASSNTAVHIEPKTGIA**

pid  **51**  **. . . . 1** **100**

1 VvCHIL 100.0% **FPPQIT---TAKPLCLLGYGITDIEIHFLQIKFTAIGVYLEPEIVGHLQP**

2 PpCHILA 42.7% **FATKFTPPTGSTELDLIGYGNTGMEIETVEIRFTAIGFYAEPSISEHLQK**

3 HlCHIL1 14.5% **FPVKLDDGKS---LN--SVGLRKKSLLGMGIKVFGFGLYADNEKLKNLLK**

pid **101**  **. . . . :** **150**

1 VvCHIL 100.0% **WKGKSGKELAEND-DFFEALISAPGEKFLRIVV-IKEIKGSQYGVQLESA**

2 PpCHILA 42.7% **WKGTPSSNLVEDDSGFHKELIQAPVEKAVRISI-IKGIKGLPYGSALQSS**

3 HlCHIL1 14.5% **LKIGKSP--AKPTEEMYQLVIDGDIGLTHKIVIAYSGLKMNMFKKAFSEA**

pid **151**  **. . . . 2** **200**

1 VvCHIL 100.0% **VRDRLAADDKYEEEEEEALEKVVE-FFQSKYFKKDSIITFHFPATSCTAE**

2 PpCHILA 42.7% **LRDRLVNNDLFEEEEEEALEKLAE-FFQPHNLPKGTNIIYHWATPSSVK-**

3 HlCHIL1 14.5% **LGESIMKLNGGRKNEE-LANKVLGPASDQIKLATGSEMEI-------SKL**

pid **201**  **. . . . :** **250**

1 VvCHIL 100.0% **IVFATEGK--EESKITVENANVVEMIKKWYLGGTRGVSPTTISALANTLA**

2 PpCHILA 42.7% **VSLSEEGKMPEDVAYTIDDAHVAEALLDLYLGEN-TITPSTLASVAEAIA**

3 HlCHIL1 14.5% **PGYVLETKVHGELASRVESELLCRAYFGIYLGEITMECYKE---SKEMFG**

pid **251**  **]** **257**

1 VvCHIL 100.0% **TELSK--**

2 PpCHILA 42.7% **A------**

3 HlCHIL1 14.5% **QSMLSLF**

**Figure S1. Multiple Sequence Alignment of PpCHIL-A, VvCHIL and HlCHIL1.** The alignment contains the following proteins: *Vitis vinifera*, VvCHIL (F6HC36); *Physcomitrella patens,* PpCHIL-A (PNR26722); *Humulus lupulus* HlCHIL1 (A0A2U7XUH7). Overall, HlCHIL1 from *Humulus lupulus* is 17% identical to VvCHIL and 18% identical to PpCHIL-A, whereas VvCHIL and PpCHIL-A are 44% identical. The MSA was generated using default parameters using the ClustalO online server (1).

**Figure S2. SDS-PAGE band quantification of a CHS-CHIL protein pull-down experiment with and without naringenin present.** Protein bands in the bound fractions were quantified using Biorad ImageLab software. The results of the quantification analysis of bound fractions (lanes 2, 4, 6, 8) are shown. The protein gel source image from Fig 4B is reused here to show how the Biorad ImageLab software was used to analyze this image for band and lane selection and quantitative analysis.

**Figure S3. SDS-PAGE band quantification of a CHS-CHIL protein pull-down experiment with NC present.** Protein bands in the bound fractions were quantified using Biorad ImageLab software. The results of the quantification analysis of bound fractions (lanes 2, 4, 6) are shown. The protein gel source image from Fig 4C is reused here to show how the Biorad ImageLab software was used to analyze this image for band and lane selection and quantitative analysis.

**Figure S4.** Quantification of the effects of naringenin on VvCHIL – strep-AtCHS binding based on native page electrophoresis results of protein pull-down experiments. The ratio of band intensities of VvCHIL to strep-AtCHS bands from bound fractions of native PAGE gel (Fig. **4** *B* , lower panel) were used to measure changes to complex formation caused by the presence of naringenin. At 4.30 mM naringenin, a band for untagged CHIL was below the threshold of detection in the strep-CHS bound fraction. The ratio of band intensities of untagged CHIL to strep-CHS is therefore undefined. The change in the ratio of VvCHIL : strep-AtCHS is similar the ratio calculated from the SDS-PAGE gel of the same experiment (shown in Fig. **4** *B* , upper panel and quantified in Fig. **4** *D* , black bars), demonstrating that the proteins used in this experiment were folded.

**Figure S5**. **Crystallographic dimers from VvCHIL and PpCHIL-A observed from X-ray structures**. The images show how the β-hairpin motif of each CHIL protein forms a crystallographic protein-protein interface in and around the ligand binding pocket for both symmetry-related molecules. **A)** Cartoon diagrams of VvCHIL (green) and a symmetry-related crystallographic dimer partner (gray). The location of the ligand binding pocket is shown by the presence of tryptophan 221 (W221), rendered as space filling. Overall, this VvCHIL crystallographic interface buries 1100 Å^2^ of surface area. **B)** Cartoon diagrams of PpCHIL-A (magenta) and a symmetry related crystallographic dimer partner (gray). The location of the ligand binding pocket is shown by the presence of leucine 188 (L188), rendered as space filling. Overall, this Pp-ACHIL crystallographic interface buries 1767 Å^2^ of surface area. Buried surface area for each interface is calculated using the “get area” function of PyMol (2).

**Figure S6.** CastP (3) models showing ligand binding cavities calculated using default parameters on the CastP web server: http://sts.bioe.uic.edu/castp. A) The potential ligand binding pocket (red) is in a structurally-similar position as the active site of related chalcone isomerase (CHI) enzymes. For VvCHIL, the 1.4 angstrom probe calculated a volume of 20.776 Å^3^. B) For PpCHIL-A, the 1.4 angstrom probe calculated a volume of 50.7 Å^3^.

**Figure S7**. **C⍺-based structural alignment of the PpCHIL-A X-ray structure (grey)** **to the AlphaFold-predicted structure for the PpCHIL-A amino acid sequence, “AF-A9T3E4-F1” (colored)**. AlphaFold is a computer program that leverages artificial intelligence (AI) to predict protein folds (4). Colors on the AlphaFold model show the per-residue confidence score (pLDDT) ranging from very high confidence (dark blue) to very low confidence (orange). The inlay shows the atomic details of a region of poor structural alignment at the β-hairpin motif, which is adjacent to a predicted ligand binding region and is therefore important for understanding function. The dotted lines (yellow) show measured distances (in angstroms) between aligned C⍺ atoms of the PpCHIL X-ray structure and AlphaFold-predicted structure. The largest measured positional difference in the C⍺-based alignment is 9.9 Å for threonine 40 (T40) and occurs in a “confident” region of the AlphaFold model. The C⍺-based structure alignment was performed using the align function of PyMol (2).

**Supporting References**

1. Sievers, F., Wilm, A., Dineen, D., Gibson, T. J., Karplus, K., Li, W., Lopez, R., McWilliam, H., Remmert, M., Söding, J., Thompson, J. D., and Higgins, D. G. (2011) Fast, scalable generation of high-quality protein multiple sequence alignments using Clustal Omega. *Molecular systems biology*. **7**, 539.
2. The PyMOL Molecular Graphics System, Version 1.8.6.2 Schrödinger, LLC
3. Tian, W., Chen, C., Lei, X., Zhao, J., Liang, J. (2018) CASTp 3.0: computed atlas of surface topography of proteins. *Nucleic Acids Res*. **46**, 363-367
4. Jumper, J., Evans, R., Pritzel, A., Green, T., Figurnov, M., Ronneberger, O., Tunyasuvunakool, K., Bates, R., Žídek, A., Potapenko, A., Bridgland, A., Meyer, C., Kohl, S.A.A., Ballard, A.J., Cowie, A., Romera-Paredes, B., Nikolov, S., Jain, R., Adler, J., Back, T., Petersen, S., Reiman, D., Clancy, E., Zielinski, M., Steinegger, M., Pacholska, M., Berghammer, T., Bodenstein, S., Silver, D., Vinyals, O., Senior, A.W., Kavukcuoglu, K., Kohli, P., and Hassabis, D. (2021) Highly accurate protein structure prediction with AlphaFold. *Nature*. **596**, 583-589.
